# Genetic polymorphisms analysis of CYP2D6 in the Uygur population

**DOI:** 10.1186/s12864-016-2719-x

**Published:** 2016-05-26

**Authors:** Xue He, Na He, Lisong Ren, Yongri Ouyang, Ning Zhang, Yini Ma, Dongya Yuan, Longli Kang, Tianbo Jin

**Affiliations:** Key Laboratory for Basic life science Research of Tibet autonomous region School of Medicine, Xizang Mingzu University, Xianyang, Shaanxi 712082 China; Key laboratory for molecular genetic mechanisms and intervention research on high altitude disease of Tibet autonomous region, School of Medicine, Xizang Mingzu University, Xianyang, Shaanxi 712082 China; National Engineering Research Center for Miniaturized Detection Systems, Xi’an, Shaanxi 710069 China; School of Life Sciences, Northwest University, Xi’an, Shaanxi 710069 China

**Keywords:** Frequency, Genetic polymorphisms, Phenotypic

## Abstract

**Background:**

This study aimed to investigate genetic polymorphisms of CYP2D6 among healthy Uygur individuals. Genetic polymorphisms of CYP2D6 could greatly affect CYP2D6 activity and lead to differences among individuals in drug efficacy or side effects. To investigate genetic polymorphisms of CYP2D6 in the Uygur population, we directly sequenced the whole gene in 96 unrelated, healthy Uygur volunteers from the Xinjiang Uygur Autonomous Region and screened for genetic variants in the promoter, intron, exons, and 3’UTR.

**Results:**

We detected 62 genetic polymorphisms of CYP2D6, 16 of which were novel SNP with three novel non-synonymous mutations detected for the first time. The allelic frequencies of CYP2D6*1, *10, *39, and *48 were 0.542, 0.156, 0.068, 0.229, and 0.073, respectively. The frequency of CYP2D6*1/*10 which decreased CYP2D6 enzyme activity was 31.3 %.

**Conclusions:**

Our results provided basic information about CYP2D6 polymorphisms, suggested that the enzymatic activities of CYP2D6 might be different within the Uygur ethnic group, and provide a basis for safer drug administration and better therapeutic treatment of Uygur individuals.

## Background

The CYP superfamily is one of the most important enzyme systems involved in the biotransformation of many endogenous and exogenous substances. Cytochrome P450 (CYP450) enzymes are essential for the metabolism of many medications. This class has more than 50 enzymes, among them CYP2D6 is one of the most significant enzymes [1]. The enzyme accounts for only a small percentage of all hepatic P450s, but its role in drug metabolism is extensively higher than its relative content [2]. CYP2D6 is an important polymorphic phase-I drug-metabolism enzyme and plays an important role in the metabolism of a variety of drugs and environmental compounds [3]. It is an important member of the cytochrome oxidase P450 enzyme system, and polymorphisms in CYP450 enzymes are responsible for observed variation in drug responses among patients of different ethnic origins. The CYP2D6 gene located at chromosome 22q 13.2 is one of the most polymorphic CYP450 genes. It contains nine exons and eight introns, has a full-length base coding sequence of 1491 bp, expresses about 497 amino acids, and is involved in the metabolism of 20–25 % of clinical prescription drugs [4]. For instance, Debrisoquine, Antidepressants,Tricyclic antidepressants, Beta-blockers et al. It has a high degree of genetic variation. To date, more than 300 variants of the CYP2D6 gene have been identified (http://www.cypalleles.ki.se), although multiple allele identified no function [5]. CYP2D6*3, CYP2D6*4, CYP2D6*5, and CYP2D6*6 are reported to be associated with decreased enzymatic activity, CYP2D6 * 4 is the most common mutants in Caucasians. About 5 % to 10 % and 1 % are slow metabolism in Caucasians and Asians respectively. Three common mutant alleles related with reduced catalytic activity, CYP2D6 * 10 were found mainly in Asians, CYP2D6*17 were found mainly in African. CYP2D6 * 41 were found in African and Caucasians. In addition to fast metabolizers, more than 5 % of Caucasians are classified as ultra-fast metabolizers, Because of multiple copies in the CYP2D6 gene which enhances the ability of metabolism [6]. For example, seven percent of Caucasoid and two to seven percent of Negroid individuals are poor metabolizers of drugs dependent on CYP2D6, which metabolizes many beta blockers, antidepressants, opioids, and other compounds [7]. The Uygur population, comprising 10,069,346 individuals (The Sixth National Census), lives almost entirely within the Xinjiang Uygur Autonomous Region, Northwestern frontier area of China, represents a typical admixture population with a genetic background of Caucasians (40 %) and East Asians (60 %) [8, 9]. At present, there is no comprehensive system for the study of CYP2D6 polymorphisms in the Uygur population of China. So to establish a database of CYP2C9 allele frequencies for the healthy Uygurs, which would be useful for personalized medicine, we decided to systematically screen the polymorphisms of the CYP2C9 gene and compared their allelic frequencies with previous data of other ethnic groups. We hoping to identify characteristics of the genetic polymorphisms of CYP2D6 and provide reasonable recommendations pertaining to the safer administration of drugs dependent on CYP2D6 in the Uygur population.

## Methods

### Study subjects

We recruited a random sample of 96 healthy, unrelated Uygur individuals (48 males and 48 females) between November 2014 and January 2015 from the Tibet Nationality College in Xianyang for genetic polymorphism research. All of the chosen subjects were volunteers living in the Xinjiang Uygur Autonomous Region of China and had at least three generations of Uygur paternal ancestry. Our research adopted strict recruitment and exclusion rules. We excluded individuals with chronic diseases, conditions involving vital organs (lung, heart, kidney, brain, and liver), and several metabolic endocrinological, metabolic, and nutritional diseases. The purpose of the exclusion procedures was to minimize the known environmental and therapeutic factors that influence genetic variation in the genes of interest.

We informed all of the participants of the experimental procedures and the purpose of the study. The Human Research Committee for the Approval of Research Involving Human Subjects at the Xizang Mingzu University approved the use of human tissue in this study. We also obtained signed, informed consent from each study participant.

### PCR and DNA sequencing

Genomic DNA was extracted from 300 μl peripheral blood using a GoldMagMini Whole Blood Genomic DNA Purification Kit (GoldMag Ltd). The purity of the extracted DNA reached above 99 %. Our PCR primers, which were described in a previous study, were designed to amplify 2000 bp of the 5’ flanking regions, all exons, and all introns of the CYP2D6 gene [10]. The 10 μl PCR system contained 5 μl Hotstar Taq Master Mix, 1 μl genomic DNA (20 ng/μl), 0.5 μl each primer pair (5 μM), and 3 μl deionized water. The PCR reaction conditions were as follows: the thermal profile consisted of denaturation at 95 °C for 15 min, followed by 35 cycles of denaturation at 95 °C for 30 s, 60 °C for 30 s, and extension at 72 °C for 1 min, followed by a final extension at 72 °C for 3 min and subsequent storage at 4 °C. We detected the PCR products by agarose gel electrophoresis and directly sequenced them using an ABI Prism BigDye Terminator Cycle Sequencing Kit version 3.1 on an ABI Prism 3100 sequencer (Applied Biosystems).

### Data analysis

The CYP2D6 variants were named based on the nucleotide reference sequence AY545216 (http:www.cypalleles.ki.se/) and the protein reference sequence P10635. The allelic and genotypic frequencies were calculated by a statistical method. Comparisons of allelic frequencies among different geographic populations or other ethnic populations were done using chi-squared tests with the significance level set at 0.05. Our study used Haploview 4.1 to assess linkage disequilibrium (LD) and Hardy-Weinberg equilibrium for each genetic variant [11]. We constructed haplotypes from selected tag SNPs and derived the haplotype frequencies for the Uygur population.

### Functional prediction

To analyze variants in the exon regions of CYP2D6, our study adopted the online tool PolyPhen2 (http://genetics.bwh.harvard.edu/pph2/) and SIFT (http://sift.bii.a-star.edu.sg/) to predict the protein function of missense mutations, which could change the function of the cytochrome P450 enzyme. Each variant was evaluated based on the impact of protein function. The PolyPhen2 output was divided into five categories: probably benign (0.000–0.999), borderline (1.000–1.249), potentially damaging (1.250–1.449), possibly damaging (1.500–1.999) and probably damaging (≥2.000). The protein function predicted by two aspects included HumVar and HumDiv.. The SIFT output was divided into four categories: tolerant (0.201–1.00), borderline (0.101–0.20), potentially intolerant (0.051–0.10) and intolerant (0.00–0.05).

## Results

### Genetic variants

We successfully identified CYP2D6 polymorphisms in 96 healthy, unrelated Uygur volunteers from the Tibet Nationality College. We identified a total of 62 CYP2D6 polymorphisms in the current Uygur population which contained 16 novel found SNP. Among 62 Uygur CYP2D6 polymorphisms, there were 19 amino-acid effects which contained nine synonymous mutations and ten non-synonymous mutations, and five of which were novel. The synonymous mutations included 2471 T > C and 3273G > C, and the non-synonymous mutations included 1673G > A, 2467 T > C and 2607G > A (Table 1).

**Table 1:** The frequencies and positions of CYP2D6 genetic variants in the Uygur population

SNP	Allele	Position	Nucleotide change	Frequency	Region	Amino-acid effect
rs1080989		−1000	R(G > A)	46.67 %	Promoter	No translated
rs28624811		−740	Y(C > T)	59.38 %	Promoter	No translated
rs28633410		−678	R(G > A)	59.38 %	Promoter	No translated
rs74966855		−498	M(C > A)	5.21 %	Promoter	No translated
rs1080992		−365	R(G > A)	4.17 %	Promoter	No translated
/		−334	S(G > C)	1.04 %	Promoter	No translated
rs34167214		−331	K(T > G)	1.04 %	Promoter	No translated
rs35534760		−328	Y(C > T)	1.04 %	Promoter	No translated
		−327	R(A > G)	1.04 %	Promoter	No translated
/		−321	S(C > G)	1.04 %	Promoter	No translated
/		−320	R(A > G)	1.04 %	Promoter	No translated
/		−203	Y(C > T)	1.04 %	Promoter	No translated
/		−202	R(G > A)	1.04 %	Promoter	No translated
/		−97	R(G > A)	1.04 %	Promoter	No translated
rs769258		31	R(G > A)	3.16 %	Exon1	Val11Met
rs1065852	CYP2D6*10	100	Y(C > T)	45.26 %	Exon1	Pro34Ser
rs146558635		123	M(C > A)	1.05 %	Exon1	Pro41 = SM
rs1080995		214	S(G > C)	59.38 %	Intron 1	No translated
rs1080996		221	M(C > A)	59.38 %	Intron 1	No translated
rs74644586		223	S(C > G)	59.38 %	Intron 1	No translated
rs76312385		227	Y(T > C)	59.38 %	Intron 1	No translated
rs75276289		232	S(G > C)	61.46 %	Intron 1	No translated
rs28695233		233	M(A > C)	45.83 %	Intron 1	No translated
rs56011157		245	R(A > G)	45.83 %	Intron 1	No translated
/		254	K(G > T)	1.04 %	Intron 1	No translated
rs29001678		270	Y(C > T)	2.08 %	Intron 1	No translated
rs28371699		310	K(G > T)	84.38 %	Intron 1	No translated
/		317	R(A > G)	1.05 %	Intron 1	No translated
/		323	R(G > A)	1.05 %	Intron 1	No translated
rs28371701		745	S(C > G)	57.29 %	Intron 1	No translated
rs71328650		842	K(T > G)	57.29 %	Intron 1	No translated
rs28371703	CYP2D6*48	973	M(C > A)	14.58 %	Exon 2	Leu91Met
rs28371704		983	R(A > G)	14.58 %	Exon 2	His94Arg
rs28371705		996	S(C > G)	16.67 %	Exon 2	Thr98 = SM
rs1081003	CYP2D6*10	1038	Y(C > T)	31.25 %	Exon 2	Phe112 = SM
rs368389952		1070	R(G > A)	1.04 %	Intron 2	No translated
rs1081004		1169	R(G > A)	4.17 %	Intron 2	No translated
rs1058164	CYP2D6*10 CYP2D6 *39	1662	S(G > C)	77.08 %	Exon 3	Val136 = SM
novel		1673	R(G > A)	1.04 %	Exon 3	Arg140His
novel		2467	Y(T > C)	7.29 %	Exon 5	Leu231Pro
novel		2471	Y(T > C)	9.38 %	Exon 5	His232 = SM
rs28371718		2576	M(C > A)	9.38 %	Exon 5	Pro267 = SM
novel		2607	R(G > A)	100.00 %	Exon 5	Glu278Lys
rs201830078		2611	W(T > A)	9.38 %	Exon 5	Met279Lys
rs76015180		2662	R(G > A)	1.09 %	Intron 5	No translated
rs28371722		2664	R(G > A)	6.52 %	Intron 5	No translated
rs187203531		2721	S(G > C)	1.09 %	Intron 5	No translated
rs28371726		3255	Y(T > C)	1.04 %	Exon 7	His361 = SM
novel		3273	S(G > C)	1.04 %	Exon 7	Giy367 = SM
/		3350	K(G > T)	1.04 %	Intron 7	No translated
rs1985842		3385	M(C > A)	58.33 %	Intron 7	No translated
rs28578778		3394	Y(T > C)	3.13 %	Intron 7	No translated
rs28371729		3436	M(C > A)	2.08 %	Intron 7	No translated
rs2004511		3583	R(A > G)	44.79 %	Intron 7	No translated
rs28371730		3585	R(G > A)	59.38 %	Intron 7	No translated
rs28371731		3791	Y(C > T)	59.38 %	Intron 7	No translated
rs28371732		3829	R(G > A)	1.04 %	Exon 8	Ser401 = SM
rs769157652	CYP2D6*27	3854	R(G > A)	13.54 %	Exon 8	Glu410Lys
rs1135840	CYP2D6*10 CYP2D6 *39	4181	S(G > C)	100.00 %	Exon 9	Ser486Thr
/		4375	Y(C > T)	1.04 %	3’UTR	No translated
rs116390392		4482	R(G > A)	59.38 %	3’UTR	No translated
rsr35028622		4723	K(T > G)	61.40 %	3’UTR	No translated

The position is according to the reference sequence AY545216 in Genbank; Not translated: this SNP has no effect on the protein sequence; UTR means untranslated region; SM means synonymous mutation

### Allelic frequencies and genotypic frequencies

We identified five CYP2D6 alleles in the Uygur population. The CYP2D6*1 allele had the highest frequency (47.4 %) and represented the wild-type CYP2D6 allele, which was followed by the CYP2D6*39 allele (22.9 %) and the CYP2D6*10 allele (15.6 %). The other two alleles, CYP2D6*27 and *48, were relatively rare. The CYP2D6 allelic frequencies within the Uygur population are shown in Table 2.

**Table 2:** Allelic frequencies of CYP2D6 in the Uygur population

Allele	Total (*n* = 192)	Phenotype	Frequency (%)
*1	91	Normal	47.4
*10	30	Decreased	15.6
*27	13	Normal	6.8
*39	44	Normal	22.9
*48	14	Normal	7.3

We also detected five CYP2D6 genotypes, with frequencies ranging from 5.2 to 35.4 % in the Uygur population. The heterozygous genotype *1/*10 (31.3 %) led to a decrease in enzyme activity, while the other four genotypes, including *1/*27 (13.5 %), *1/*39 (35.4 %), *1/*48 (14.6 %), and the rare homozygous *39/*39 (5.2 %), did not affect the enzyme activity. According to the Haploview analysis, all of the allelic and genotypic frequencies fit Hardy-Weinberg equilibrium. The CYP2D6 genotypic frequencies are shown in Table 3.

**Table 3:** Genotypes of CYP2D6 in the Uygur population

Genotype	Total (*n* = 96)	Phenotype	Frequency (%)
*1/*10	30	Decreased	31.3
*1/*27	13	Normal	13.5
*1/*39	34	Normal	35.4
*1/*48	14	Normal	14.6
*39/*39	5	Normal	5.2

We further compared the CYP2D6 allelic frequencies between the Uygur population and other ethnic populations from various countries. The frequencies of CYP2D6*10, *27, and *39 in the Uygur population were different from those in the other ethnic groups (Table 4).

**Table 4:** CYP2D6 allelic frequencies in the Uygur population

Population	Total number	CYP2D6 frequency (%)				Reference
		*1	*10	*27	*39	
Uygur	96	47.4	15.6	6.8	22.9	
UAE	151	39.1	3.3**	0.7**	4.0**	[28]
Korean	400	33.25**	45.0**	0.38**	0.63**	[29]
Japanese	206	43.0	38.1**	0.2**	0.3**	[30]
Brazilian	873	39.9	2.05**	0	0.8**	[31]
Chinese Han	400	24.65**	52.53**	0	0	[10]
Caucasian	330	35.5*	2.7**	0	0	[32]
Spanish	105	31*	1.9**	0	0	[33]
Austrian	93	34.9	4.3**	0	0	[34]
Sardinian	250	31.4**	5.4**	0	0	[35]

Note: **p < 0.01; *p < 0.05; the p value means the comparison of the allelic frequencies between the Uygur population and other populations

### Linkage disequilibrium analysis

**Fig. 1:**
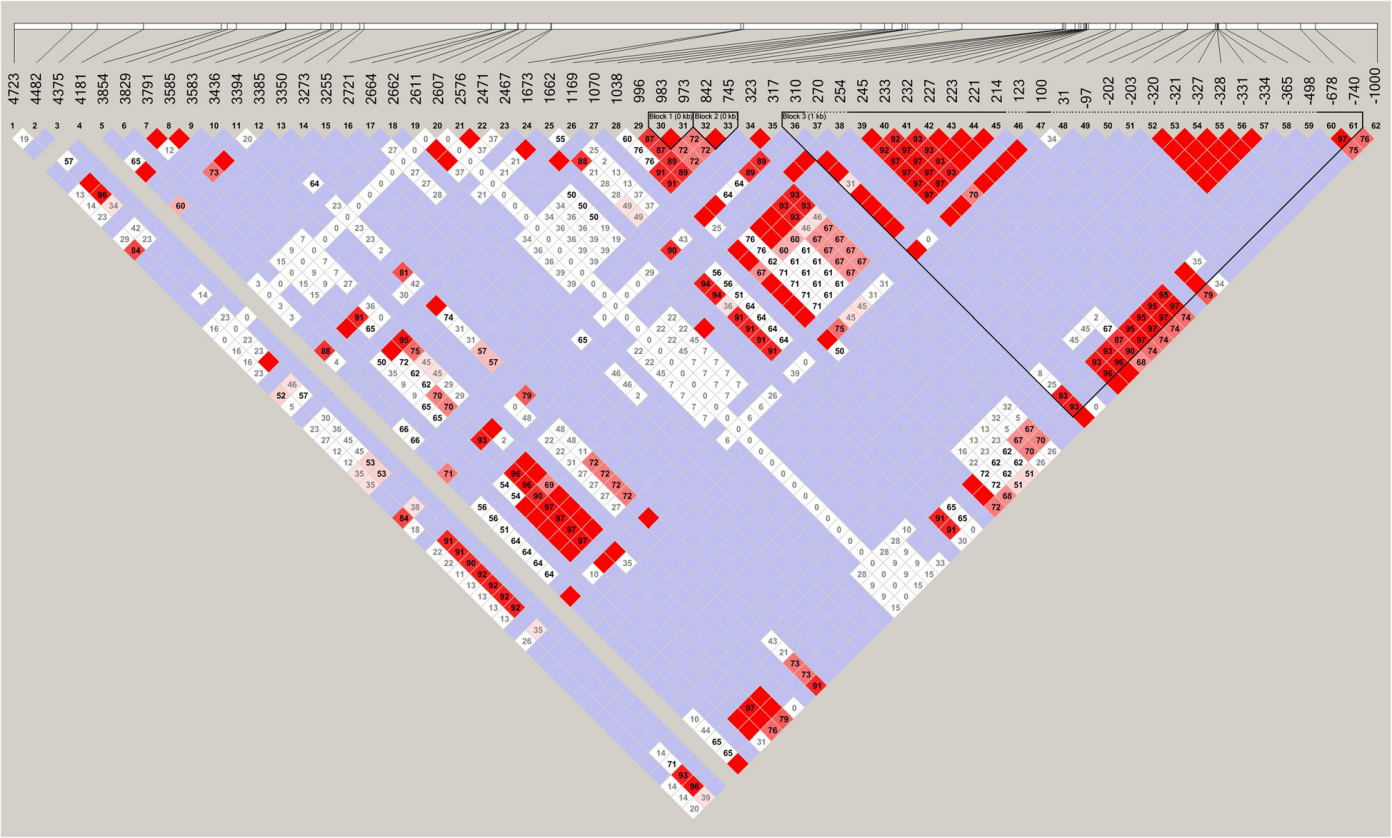
Linkage disequilibrium analysis of CYP2D6 genetic polymorphisms. Strong LD is displayed by *bright red* (very strong: LOD > 2, D’ = 1) or *pink red* (moderately strong: LOD > 2, D’ < 1), intermediate LD is displayed by *blue* (LOD < 2, D’ = 1), and absence of LD is displayed by *white* (LOD < 2, D’ < 1)

We adopted the Haploview software to perform LD analysis with confidence intervals to define blocks. LD is the population-genomic feature used in genetic association studies to find the location of variants that predispose individuals to genetic diseases [12]. The D’ value indicated the extent of LD between these SNPs which shown in Fig. 1. We identified two LD blocks from our 62 Uygur polymorphisms. The first block included two very tightly correlated markers: 842 T > G and 745C > G. The second block had a very strong linkage among 310G > T, 245A > G, 233A > C, 232G > C, 227 T > C, 223C > G, 221C > A, 214G > C, 100C > T,−678G > A and−740C > T.

### Non-synonymous mutation effects

**Fig. 2:**
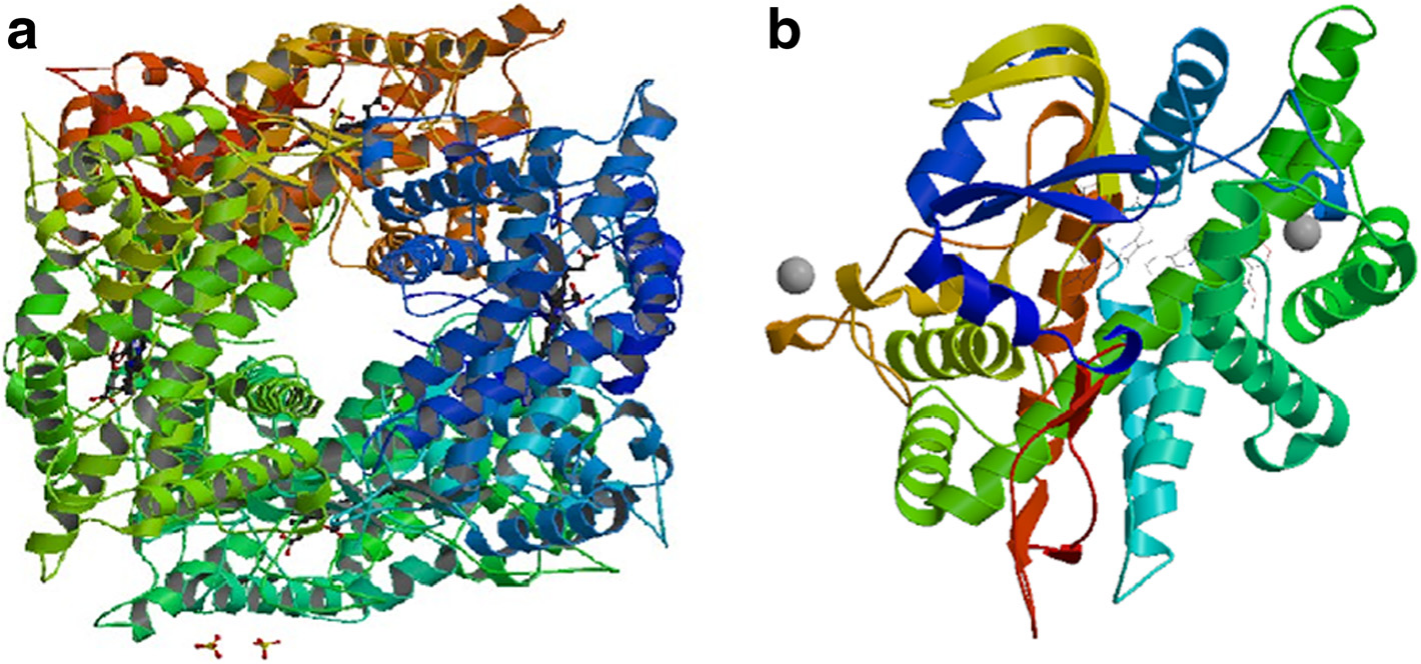
Predicted computationally construct the structure of mutant protein. **a** Crystal Structure of Human Cytochrome P450 2D6. **b** CYP2D6*10

**Fig. 3:**
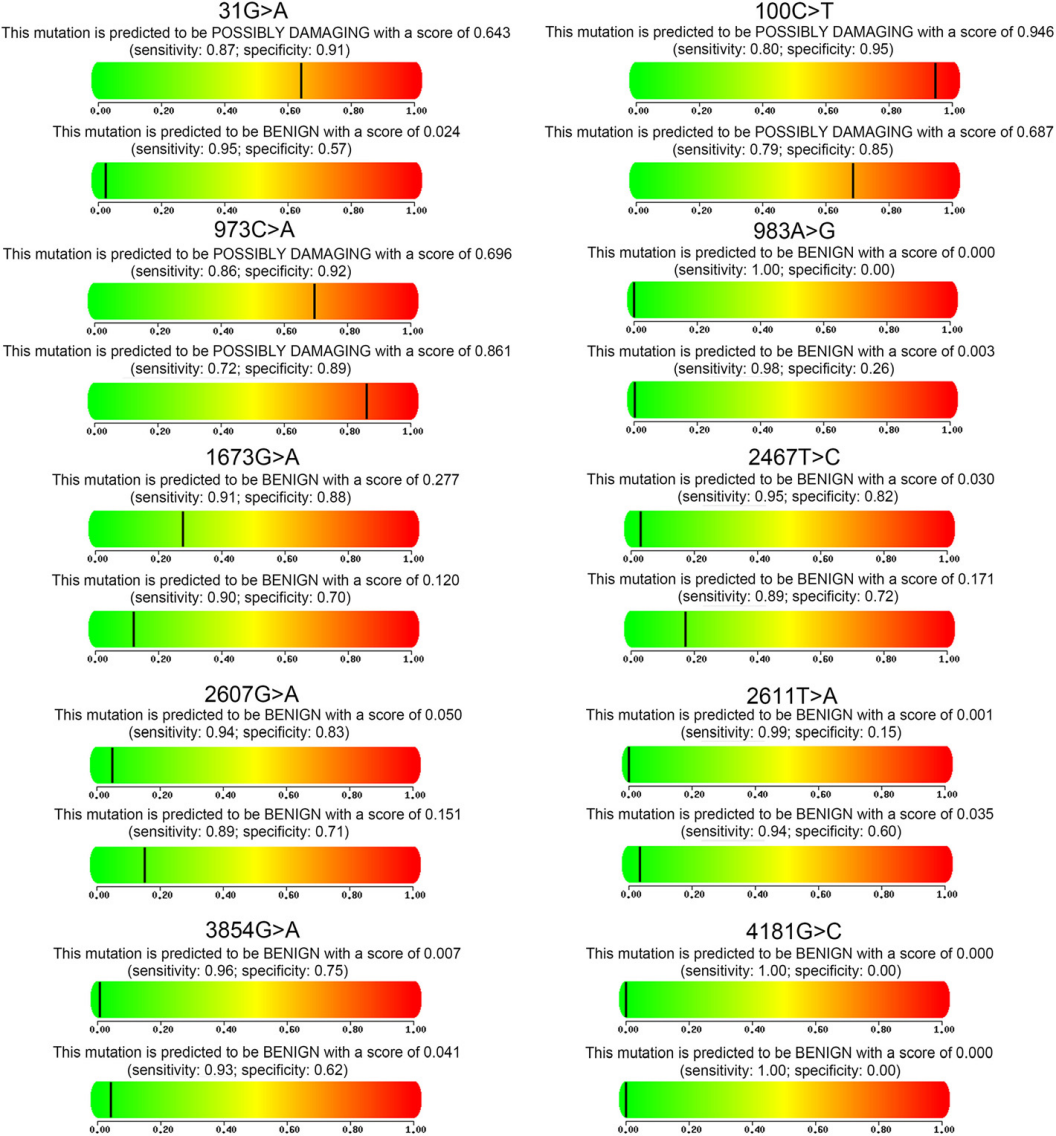
Predicted protein function of all the CYP2D6 nonsynonymous mutations

The functional consequence of the novel non-synonymous mutations be predicted by computationally construct the structure of mutant protein in Fig. 2. We used PolyPhen-2 and SIFT to predict the function of CYP2D6 which carried the ten different non-synonymous mutations. First, we verified CYP2D6*10 (100C > T) is the mutant with possibly damaging the enzyme activity and CYP2D6*48 (973C > A) is another. Then we predicted the protein function of 3 novel amino-acid change and found 1673G > A, 2467 T > C and 2607G > A is benign to the protein function. From the result of SIFT, we found that the results is not consistent with PolyPhen-2’. The results of SIFT shown in Table 5 and Fig. 3 shown the predicted results from PolyPhen-2.

**Table 5:** Results of SIFT predictions of non-synonymous SNPs

SNP	Substitution	dbSNP	Score	Prediction
31G > A	Val11Met	rs769258	0.12	TOLERATED
100C > T	Pro34Ser	rs1065852	0.00	AFFECT PROTEIN FUNTION
973C > A	Leu91Met	rs28371703	0.01	AFFECT PROTEIN FUNTION
983A > G	His94Arg	rs28371704	0.35	TOLERATED
1673G > A	Arg140His	novel	0.00	AFFECT PROTEIN FUNTION
2467 T > C	Leu231Pro	novel	0.11	TOLERATED
2607G > A	Glu278Lys	novel	0.06	TOLERATED
2611 T > A	Met279Lys	rs201830078	0.00	AFFECT PROTEIN FUNTION
3854G > A	Glu410Lys	rs769157652	0.14	TOLERATED
4181G > C	Ser486Thr	rs1135840	0.37	TOLERATED

## Discussion

The CYP2D6 gene has been considered very difficult for genotypic analysis, because of its numerous polymorphisms including SNPs, gene deletions, and duplications. To date, multifarious studies have analyzed CYP2D6 genetic polymorphisms. Baclig et al. found that the allelic frequencies of CYP2D6*10 among healthy Filipino volunteers were similar to those among other Asians but markedly different from those among Caucasian populations [13]. We determined that CYP2D6*10, the most prevalent allele reported in the Asian population, had a frequency of only 15.6 % among Uygur individuals. Zuo et al. found that the frequency of the CYP2D6*10 allele was not significantly different among the Han, Mongolian, or Hui populations; although the Uighur population showed significantly lower frequencies of that allele compared with the other three populations [14]. Several drugs, such as hydrocodone, are metabolized by cytochrome 450 enzymes [15], so we recommended that the dosage of that drug for Uygur individuals should be less than that used for members of the other three populations.

There were marked differences in the CYP2D6 allelic frequencies among populations with different continental origins. Some alleles were observed at high frequencies in different populations, such as CYP2D6*4 in Europeans and CYP2D6*17 in Africans [16, 17]. For the analysis of the genetic variants of CYP2D6 in the Uygur population, there were 19 mutations, comprising nine synonymous mutations and ten missense mutations. Six novel missense mutations were located at the transcription site and were predicted to result in a loss of enzyme function. Only one novel mutation was located in the 3’UTR and was therefore not translated, and we did not find any mutations in the promoter region. The CYP2D6*27 allelic frequency was 6.8 % in the Uygur population; its isoforms all showed active codeine metabolism and dextromethorphan demethylation [18]. We also found a high frequency of CYP2D6*39 (22.9 %) in the Uygur population, which was different from that in the other ethnic groups and had not been previously described in the Uygur population. The CYP2D6*39 allele was previously reported to be common in Asian populations and to possibly decrease the expression level of the CYP2D6 protein; however, that allele could not transform the protein function [19].

To date, more than 50 clinically important drug substrates of CYP2D6 have been reported [20], including codeine [17], dextromethorphan [21], cyclophosphamide [22], tamoxifen [23], and ethylmorphine. There has been a lack of research to provide information regarding the influence of CYP2D6*10 on the metabolic activity of CYP2D6 in the Uygur population [24]. Hamzeiy et al. [25] determined that CYP2D6*10 occurred more frequently in Iran (9 %) than in the UAE (3.3 %). Our study provides new data on CYP2D6 gene polymorphisms in the Uygur population and compares the frequencies of polymorphisms between the Uygur population and other ethnic populations. The differences in allelic frequencies indicate that the genetic composition also varies between the different geographical populations. That variation could contribute significantly toward a better understanding of CYP2D6 polymorphisms and to the development of a database for personalized medicine in the Uygur population. Britzi et al. [26] found a significant difference in the distribution of the metabolic ratio of the “extensive metabolizer” phenotype among Ethiopian, Russian, and Yemenite populations.

We also investigated the haplotype and LD pattern construction of the CYP2D6 gene in the Uygur population. The LD provided a basic profile of the genomic structure of CYP2D6 in the Uygur population. Our study analyzed the pattern of LD in CYP2D6 among the Uygur and identified two blocks. We chose to deselect the loci that did not fit Hardy-Weinberg equilibrium to ensure that our further analyses produced reliable results. In order to see the differences in the LD structure, we constructed the haplotypes from the tag SNPs, so the haplotype structure and distributions were different in different populations. The combined genotypic effects of some decreased-function variants would result in inactive enzymes. Combinations of different polymorphisms might produce markedly different results in terms of CYP2D6 activity.

There are some limitations in our study must be noted. The sample size of this study is relatively small. The number of patients in several genotypic groups was small when the samples were divided into different groups according to the genotype, which could influence the study results. However, the current study possessing enough power.

Overall, we determined that about 30 % of Uygur individuals had genotypes associated with decreased enzyme activity, while about 70 % of the people in our research had genotypes associated with normal enzyme activity. A previous study found some relationships among haplotypes and certain diseases, drug clearance rates, and adverse drug reactions, involving several joint mutation sites that reduced the functions of the enzyme [27]. Different polymorphic sites and interactions led to significant differences in enzyme activity, and haplotype analysis was more beneficial to the identification of metabolic phenotypes.

## Conclusion

In summary, our research systematically analyzed the variants of CYP2D6 by directly sequencing that gene in members of the Uygur population and comparing the results with those from other ethnic populations around the world. Our work offers some useful information for the establishment of a database of CYP2D6 genetic polymorphisms in the Uygur population, which would provide a theoretical basis for individualized medical treatment and drug genomics studies. Next, we will concentrate on identifying CYP2D6 variants in a larger sample size of Uygur individuals in order to benefit the advancement of personalized medicine.

## Abbreviations

CYP: Cytochrome P450
LD: Linkage disequilibrium
